# Genetic and life‐history changes associated with fisheries‐induced population collapse

**DOI:** 10.1111/eva.12060

**Published:** 2013-02-25

**Authors:** Lilian Pukk, Anna Kuparinen, Leili Järv, Riho Gross, Anti Vasemägi

**Affiliations:** ^1^ Department of Aquaculture Estonian University of Life Sciences Estonia; ^2^ Department of Environmental Sciences University of Helsinki Finland; ^3^ Ecological Genetics Research Unit Department of Biosciences University of Helsinki Finland; ^4^ Estonian Marine Institute University of Tartu Estonia; ^5^ Department of Biology University of Turku Finland

**Keywords:** Eurasian perch (*Perca fluviatilis* L.), fisheries‐induced evolution, population collapse, population genetics, population replacement, temporal trend

## Abstract

Over the recent years, growing number of studies suggests that intensive size‐selective fishing can cause evolutionary changes in life‐history traits in the harvested population, which can have drastic negative effects on populations, ecosystems and fisheries. However, most studies to date have overlooked the potential role of immigration of fish with different phenotypes as an alternative plausible mechanism behind observed phenotypic trends. Here, we investigated the evolutionary consequences of intensive fishing simultaneously at phenotypic and molecular level in Eurasian perch (*Perca fluviatilis* L.) population in the Baltic Sea over a 24‐year period. We detected marked changes in size‐ and age‐distributions and increase in juvenile growth rate. We also observed reduction of age at sexual maturity in males that has frequently been considered to support the hypothesis of fisheries‐induced evolution. However, combined individual‐based life‐history and genetic analyses indicated increased immigration of foreign individuals with different life‐history patterns as an alternative mechanism behind the observed phenotypic change. This study demonstrates the value of combining genetic and phenotypic analyses and suggests that replacement or breakdown of locally adapted gene complexes may play important role in impeding the recovery of fish populations.

## Introduction

Fishing causes substantial mortality in commercially exploited fish stocks, often exceeding the level of natural mortality (Stokes and Law 2000; Jørgensen et al. 2007; Law 2007). As a consequence, over 80% of the world's fish species are currently fully exploited or overfished (FAO 2011). Moreover, comparison of current population abundance estimates with historical records indicates that heavy exploitation has caused drastic collapses in many commercially harvested marine stocks (Pauly et al. 2002; Myers and Worm 2003; Hutchings and Reynolds 2004; Pauly 2008). Despite various management strategies aimed at reducing fishing pressure, only few populations show signs of recovery in abundance (Stockwell et al. 2003; Hutchings and Reynolds 2004). This suggests that exploitation, and subsequent population recovery, is not simple processes decreasing or increasing biomass, but several environmental, ecological and genetic mechanisms can be involved, such as habitat alterations, shifts in species interactions and changes in population genetic composition as well as the evolution of life‐history traits (Hutchings 2005; Enberg et al. 2009; Swain 2011; Kuparinen and Hutchings 2012). Nonetheless, little remains known about the genetic consequences of harvesting, partly because temporal investigations that simultaneously evaluate phenotypic and molecular changes are very scarce (Árnason et al. 2009; Jakobsdóttir et al. 2011).

Over the last decade, increasing numbers of studies of experimental and wild harvested fish populations have reported significant temporal shifts in life‐history traits that have been associated with fisheries‐induced selection (Conover and Munch 2002; Jørgensen et al. 2007; Kuparinen and Merilä 2007; Allendorf et al. 2008; Allendorf and Hard 2009). At the same time, many studies have also pointed out the difficulties in distinguishing phenotypical responses from true evolutionary changes (Stokes and Law 2000; Kuparinen and Merilä 2007; Law 2007; Kuparinen and Merilä 2008). For example, faster growth and earlier maturation at a smaller size can be a consequence of fisheries‐induced evolution (FIE) or, alternatively, may be explained by a decline in fish density, where reduced competition for food and space leads to increased growth and earlier maturation (Law 2000). Furthermore, only a few of the studies focusing on disentangling genetic and environmental effects of fisheries exploitation have explicitly tested whether the observed phenotypic shifts could be at least partly attributable to population replacements, immigration or emigration (Jakobsdóttir et al. 2011; Hansen et al. 2012). However, while the role of population replacement is a relatively unconsidered concept in fisheries literature, this is not the case for other taxa (e.g. Pergams and Lacy 2008; Mackie et al. 2010; Saltonstall 2002). For example, rapid morphological and genetic changes of white footed mice (*Peromyscus leucopus*) have been associated with immigration of non‐native individuals with a distinct phenotype that displaced the previously resident population due either to selective advantage in the changing habitat or to genetic swamping (Pergams and Lacy 2008). Nevertheless, given that the majority of fish species consist of reproductively isolated populations and genetic differences often exist at a much smaller scale than the dispersal ability of the species (Allendorf et al. 2008; Hauser and Carvalho 2008; Bergek and Björklund 2009), the influx of non‐native individuals with different geno‐ and phenotypes could be more widespread than previously anticipated. For instance, temporal genetic analysis of North Sea cod (*Gadus morhua*) demonstrated that a heavily exploited population can be strongly affected by gene flow from neighbouring populations (Hutchinson et al. 2003). Similarly, drastic changes in population genetic composition have been well documented in many fish species, particularly in systems with prolonged and/or strong anthropogenic impacts (Vasemägi et al. 2005; Nielsen and Hansen 2008, Palstra and Ruzzante 2010) or seasonal variations in abiotic conditions (Crispo and Chapman 2009).

**Figure 1:**
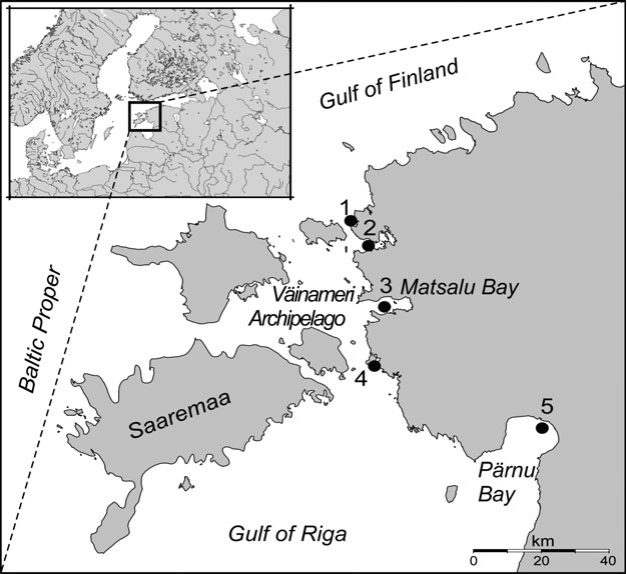
Sampling locations of perch in Väinameri (Moonsund) Archipelago area (Paslepa (1); Haapsalu (2); Matsalu Bay (3); Virtsu (4)) and Pärnu Bay (5) in the Baltic Sea.

**Figure 2:**
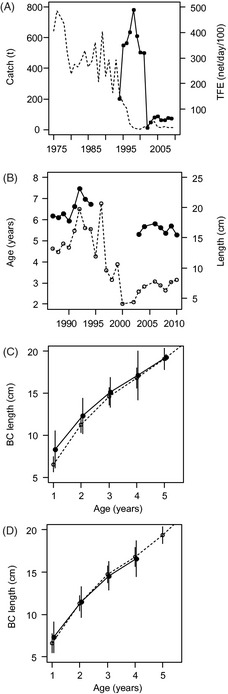
Catch statistics of perch (*Perca fluviatilis* L.) in Väinameri (Moonsund) area and changes of life‐history traits in Matsalu Bay. (A) Official catch of perch in Matsalu Bay from 1975 to 2010 (dashed line) and the total fishing effort, TFE (net/day/100) of gill net fishery from 1994 to 2009 (filled circles); (B) mean age and length of perch from 1987 to 2010 (empty and filled circles respectively); (C) length‐at‐age estimates back‐calculated (BC length) from scales; (D) same as figure (c), but individuals with non‐native genotype were excluded from the analysis. Full and open circles represent post‐ (2009–2010) and pre‐collapse (1987–1990) length‐at‐age respectively. Standard deviations are encompassed by vertical lines.

Here, we investigated the consequences of overexploitation and subsequent population recovery of the Eurasian perch (*Perca fluviatilis* L.) population in Matsalu Bay, in the eastern Baltic Sea (Fig. 1), based on combined temporal genetic and life‐history analyses over a 24‐year period. The system provided a unique opportunity for studying the effects of intensive fishing as prior to the 1990s, when Estonia was still part of the Soviet Union, only commercial fishermen had rights to fish in the study area with trap and gill nets. However, after the Republic of Estonia reclaimed its independence from the Soviet Union in 1991, fishing became virtually unregulated for a short period of time in many coastal areas of Estonia, which encouraged overexploitation and lead to the subsequent collapse of the Matsalu Bay perch population in the late 1990s (Fig. 2A; Järv 2002; Järv et al. 2005). Despite multiple fishing regulations that were introduced in 1999, the Matsalu Bay perch population has not recovered and the population size has remained very low (Järv et al. 2005).

## Materials and methods

### Study area and sample collection

Matsalu Bay in the Väinameri (Moonsund) Archipelago is situated in the north‐eastern part of the Baltic Sea (Fig. 1) and is very shallow (a mean depth of 1.5 m) with a surface area of 67 km^2^ (Kotta et al. 2008). The water chemistry parameters are determined by the mixing of water from the Kasari River (the mean yearly inflow of 40‐50 m^3^/s), and the Väinameri area and its salinity varies between 2 and 5 ‰. The shallow areas of Matsalu Bay are especially good spawning places for the Eurasian perch, providing suitable food for juveniles and adults (Järv 2000). Prior to the 1990s, the annual official catch of perch in the Väinameri area was approximately 500 metric tonnes, while officially recorded catches today are below 20 tonnes (Fig. 2A; Järv et al. 2000; Järv 2002; Järv et al. 2005). Based on previous studies, Eurasian perch show strong site fidelity (Bergek and Björklund 2009) and seldom migrate more than 10 km (Saulamo and Neuman 2002). In Matsalu Bay, for example, 58.2% of the tagged perch have been recaptured within a 10 km area from the place of release (Järv 2000).

Perch samples were collected during the spawning season when the water temperature reached 6–8 °C in 1987–2010 in Matsalu Bay (sampling date: median, 22_nd_ of May; range, from 15_th_ of April to 28_th_ of June) and neighbouring regions (Pärnu Bay and coastal areas near Haapsalu, Paslepa and Virtsu) using fyke nets (mesh size 16 and 18 mm). For phenotypic analyses, information was extracted from 1 570 individuals (Matsalu Bay: years 1987, *n* = 102, 1988, *n* = 100, 1989, *n* = 102, 1990, *n* = 100, 1991, *n* = 99, 1992, *n* = 102, 1993, *n* = 102, 1994, *n* = 101, 1995, *n* = 101, 1996, *n* = 101, 1997, *n* = 101, 1998, *n* = 99, 1999, *n* = 56, 2000, *n* = 7, 2002, *n* = 22, 2003, *n* = 12, 2004, *n* = 12, 2006, *n* = 17, 2007, *n* = 28, 2008, *n* = 33, 2009, *n* = 73, 2010, *n* = 100) (standard length, maturity status, sex), and ages and growth histories of these individuals were obtained through back‐calculations from operculum and scale samples (Pierce et al. 1996). For DNA analyses, dried scale or opercular bone and fin clip (Pärnu Bay, 2010, n = 64) samples (*n* = 1 088) were selected (Matsalu Bay: years 1987, *n* = 104, 1988, *n* = 201, 1990, *n* = 100, 1993, *n* = 102, 1996, *n* = 102, 2000, *n* = 76, 2009, *n* = 73 and 2010, *n* = 100; Pärnu: 1987, *n* = 52, 2010, *n* = 52; Haapsalu: 2010, *n* = 19; Paslepa, 2010: *n* = 19 and Virtsu, 2010: *n* = 24). For subsequent population genetic analyses, all individuals were divided into cohorts according to their year of birth (Table 1).

**Table 1:** Temporal and spatial information of Eurasian perch samples used for genetic analysis

Sampling site	Cohort/sampling year^a^	*n*	*A*	*A*_r_	*H*_e_	*H*_o_	H–W^c^	LD	*M*‐ratio
Matsalu Bay	≤1981	26	5.57	3.46	0.50	0.52	NS	0	0.49
Matsalu Bay	1982	63	5.71	3.15	0.48	0.47	NS	0	0.51
Matsalu Bay	1983	67	6.86	3.35	0.51	0.52	NS	0	0.60
Matsalu Bay	1984	52	5.71	3.26	0.50	0.47	NS	0	0.50
Matsalu Bay	1985	48	5.00	3.22	0.50	0.52	NS	0	0.45
Matsalu Bay	1986	52	6.29	3.24	0.50	0.45	NS	0	0.53
Matsalu Bay	1987	57	5.86	3.34	0.51	0.46	NS	0	0.53
Matsalu Bay	1988	44	6.14	3.51	0.54	0.49	NS	1	0.54
Matsalu Bay	1989	31	6.14	3.59	0.55	0.51	NS	0	0.53
Matsalu Bay	1990	40	6.43	3.3	0.52	0.53	NS	0	0.55
Matsalu Bay	1994–1996^b^	47	5.86	3.44	0.53	0.45	NS	0	0.56
Matsalu Bay	2006	84	5.57	2.91	0.44	0.40	***	8	0.48
Matsalu Bay	2007	47	5.57	3.29	0.5	0.46	***	10	0.52
Matsalu Bay	2008	26	4.86	3.32	0.55	0.39	***	7	0.47
Pärnu Bay	1987	27	4.71	3.31	0.51	0.55	NS	0	0.45
Pärnu Bay	2010	101	8.71	3.61	0.56	0.56	NS	1	0.69
Haapsalu	2010	19	5.43	3.63	0.55	0.56	NS	0	0.46
Paslepa	2010	16	3.71	3.01	0.49	0.51	NS	0	0.35
Virtsu	2010	18	5.14	3.61	0.57	0.49	NS	0	0.44

a Matsalu Bay samples are shown as cohorts, other areas as sampling years.

b This sample consists of fish born in 1994 (*n* = 15), 1995 (*n* = 17), 1996 (*n* = 15).

c *P*‐values for Hardy–Weinberg equilibrium test after Bonferroni corrections (*k* = 133). NS denotes a non‐significant and *** highly (*P *<* *0.001) significant *P*‐value.

*n* – number of fish successfully genotyped; *A* – mean number of alleles; *A*_r_ – allelic richness.

LD – number of locus pairs (out of 21 comparisons) deviating from gametic phase equilibrium after Bonferroni corrections (*k* = 0.002).

### DNA extraction and microsatellite genotyping

Total DNA from dried scale and operculum samples was extracted following a salt‐based protocol combined with a vacuum‐based extraction method as described by Swatdipong et al. (2010a). For ethanol‐preserved samples (Pärnu Bay, 2010), DNA was isolated from fin clips according to Laird et al. (1991). Genetic diversity was initially assessed using 11 microsatellite markers: Svi6, Svi18 and Svi33 (Borer et al. 1999) and PFE01, PFE03, PFE11, PFE12, PFE14, PFE15, PFE19 and PFE22 (Zhan et al. 2009). Data for four loci were subsequently excluded due to low levels of polymorphism (Svi33, PFE14, PFE22) or due to poor amplification (PFE15).

Microsatellite loci were amplified by single‐ or multiplex polymerase chain reaction (PCR) using a three primer system (fluorescently labelled universal M13 primer, M13‐tailed forward primer and reverse primer) according to Boutin‐Ganache et al. (2001). The 6 μl PCR reaction consisted of ca. 100 ng of template DNA, 2x multiplex PCR master mix (Qiagen), 1 μM of FAM, VIC, NED or PET‐labelled universal M13 primer, 0.05 μM of forward and 0.2 μM of reverse primer. Amplifications were carried out in a PTC100 Thermal cycler (MJ Research) for PFE01 (NED), PFE12 (PET), PFE19 (PET) and in a 2720 Thermal cycler (Applied Biosystems) for Svi6 (VIC), PFE11 (VIC), PFE03 (FAM), Svi33 (FAM) with an initial heat‐activation at 95 °C for 5 min followed by 15 cycles of denaturation at 94 °C for 30 s, annealing at 55 °C (PFE12, PFE19), 53 °C (Svi6, Svi33, PFE03, PFE11) or 52 °C (PFE01) for 90 s and extension at 72 °C for 60 s. This was followed by 25 cycles of denaturation at 94 °C for 30 s, annealing at 52 °C (Svi6, Svi33, PFE03, PFE11, PFE12, PFE19) or 50 °C (PFE01) for 90 s and extension at 72 °C for 60 s. The PCR was terminated after 15 min of extension at 60 °C.

PCR products (FAM and VIC‐labelled fragments 1 μl; NED and PET‐labelled fragments 2 μl) were diluted in 100 μl of ddH_2_O, denatured and electrophoresed on an ABI Prism 3130xl genetic analyzer (Applied Biosystems/Hitachi) along with GeneScan 600 LIZ size standard (Applied Biosystems). DNA fragments were genotyped using GeneMarker 1.60 (Soft Genetics). All genotypes were manually inspected.

DNA was successfully extracted and amplified from 79.5% of the tested samples (*n* = 865; Table 1). Altogether 223 samples of 1 088 (20.5%) showed signs of contamination (the occurrence of more than two alleles per locus), or did not result in successful amplification, and were excluded from subsequent analysis. To evaluate genotyping error rate, 96 individuals (9.9% of all individuals) were re‐amplified and re‐genotyped (years 1987, *n* = 12, 1988, *n* = 14, 1990, *n* = 7, 1993, *n* = 7, 1996, *n* = 8, 1999, *n* = 11, 2002, *n* = 7, 2009, *n* = 15 and 2010, *n* = 15). Estimated allelic error rates at each locus (Pompanon et al. 2005) varied between 0% (PFE19) and 5.7% (PFE11) with a mean of 2.5%. At least one locus mismatch was detected in 7.29% of individuals and in 2.5% of alleles. According to Pompanon et al. (2005), microsatellite genotyping error rates of between 0.5 to 1% per locus are common, but also higher error rates have been reported (Taberlet et al. 1996; Ellis et al. 2011), especially when using low quality DNA (Haaland et al. 2011).

### Phenotypic analysis

Possible changes in fish life‐histories were investigated by comparing life‐history data of perch from the pre‐ and post‐collapse periods in Matsalu Bay. Growth data were available only for the pre‐collapse years 1987 and 1990 and for the post‐collapse years 2009 and 2010. In these two year groups, changes in age‐specific lengths were investigated using linear mixed effect models (LME), where sex and a factor indicating whether the individual belonged to the ‘pre‐’ or ‘post‐’ collapse group, as well as their interactions, were set to fixed effects and the cohort was set to a random effect. These analyses were conducted separately for ages from 1 to 5 years, as no older individuals were present in the post‐collapse period, and thus comparison between pre‐ and post‐collapse groups was not possible.

Maturity data were available for the pre‐collapse years 1987–1992 and post‐collapse years 2006–2008 and 2010. Sex‐specific maturity ogives (i.e. the age‐specific probability of being mature) in pre‐ and post‐collapse years were investigated through a generalized linear model (GLM) with a binomial error structure, having age, length, collapse indicator (factor, similar to that described above) as well as their two‐way interactions and the quadratic effects of age and length as effects. Model simplifications were made by stepwise model reduction based on either likelihood ratio tests (LME) or chi‐squared tests (GLM) as suggested by Crawley (2007). Whole life‐history analyses were conducted using R 2.11.1 (R Development Core Team 2010).

### Population genetic analysis

GenePop 4.0 (Rousset 2008) was used to test for deviations from the gametic phase and Hardy–Weinberg (*HW*) equilibria (5 000 iterations each). To assess levels of genetic diversity over time, allelic richness (*A*_r_; the effect of different sample sizes are taken into account), mean number of alleles (*A*), observed (*H*_o_) and expected heterozygosity (*H*_e_) were calculated using FSTAT 2.9.3.2 (Goudet 1995) and Microsatellite Toolkit 3.1.1 (Park 2001). To identify potential footprints of genetic bottleneck, the M‐ratio measure was calculated following Garza and Williamson (2001). The standard deviation of the M‐ratio was calculated by bootstrapping over loci (10 000 replications) as described in Swatdipong et al. (2010b) using the program PopTools 2.7.5 (Hood 2006).

To investigate genetic differentiation between year classes, pairwise *F*_ST_ values were calculated following Weir and Cockerham (1984) and implemented in FSTAT 2.9.3.2 (Goudet 1995). The significance of allele frequency differences between cohorts was estimated using the genetic differentiation test implemented in GenePop 4.0 (Rousset 2008).

The program Structure 2.3 (Pritchard et al. 2000) was used to detect changes in genetic composition over the period of 24 years based on multilocus genotype information. This approach aims to divide the genotype data into homogenous genetic clusters (*K*) that are in HW and gametic phase equilibrium. Twenty independent runs were performed for each value of *K* (*K* = 1–6), using prior population information, as suggested by Hubisz et al. (2009), with a burn‐in period of 100 000 iterations and 100 000 replications. The method developed by Evanno et al. (2005) was used to estimate the most likely number of clusters based on the rate of change in the logarithmic probability of data between successive *K*‐values. In addition to Matsalu Bay samples, individuals from nearby regions (Pärnu Bay and Moonsund Archipelago Sea) were also included in the analysis. To avoid spurious clustering of the individuals due to incomplete genotype data, individuals with more than two missing loci were excluded from this analysis (693 individuals retained).

The effective population size (*N*_e_) and immigration rate (*m*) of the Matsalu Bay perch population were estimated using a maximum‐likelihood method implemented in the program MLNE 1.0 (Wang and Whitlock 2003). This method requires a user‐specified upper limit for *N*_e_, which was set at 2 000 after checking that similar results were obtained with higher upper limits for *N*_e_. To obtain estimates of *N*_e_ and *m* for the pre‐ and post‐collapse periods the data were divided into two groups (from cohorts 1981 to 1990 and from cohorts 1995 to 2008, respectively). We subsequently created a putative donor population (source of immigrants) by pooling all the individual genotypes that clustered together with more than a 0.8 probability as ‘foreign’ genotypes, identified by Structure (cohorts 1987, *n* = 3; 1999, *n* = 1; 2005, *n* = 6; 2006, *n* = 41; 2007, *n* = 20; 2008, *n* = 7). We also carried out analyses using samples from neighbouring regions (Pärnu, Virtsu, Paslepa and Haapsalu Bay) as potential source populations (data not shown). The generation length for pre‐ and post‐collapse cohorts was set at 5.3 and 3.3 years, respectively, according to the mean ages of mature individuals. As the intervals between year classes were not integers, all estimates were adjusted according to the equations provided by Wang and Whitlock (2003). More specific estimations of *N*_e_ and *m* were also carried out, keeping pre‐ and post‐collapse samples separate. For pre‐collapse analysis, the 1981 and 1982 samples were pooled together (0 generation) and the subsequent year classes were analysed separately until 1990 (1.7 generations). Similarly, for post‐collapse analysis the 1989 and 1990 samples were pooled together (0 generation) and the subsequent year classes were analysed separately until 2008 (4.76 generations).

### Analysis of life‐history traits based on multilocus genotype information

To test whether the individuals belonging to distinct genetic clusters identified by Structure differed in their growth rate, the age‐specific lengths (ages from one to five) were used in linear mixed models, having sex, cluster, and their interaction as fixed effects, and cohort as a random effect. Similar to the analysis described earlier, model simplification was made by stepwise model reduction based on likelihood ratio tests (LME) using R 2.11.1.

## Results

### Changes in growth rate and maturation

The mean age and length of perch in the Matsalu Bay population decreased considerably during the study period (Fig. 2B). In the pre‐collapse years (1987–1992), the mean age and length of perch were 5.1 years and 195.3 mm respectively. However, in the post‐collapse years, the mean ages and lengths were considerably reduced (mean age 3.0 years; mean length 164.7 mm). While such a pattern can result from a shift in the demographic structure of the population, analysis of the age‐specific length showed that the growth rates of individuals had changed over time. One‐year‐old perch in 2009 and 2010 were on average 17.5 mm longer (Fig. 2C) (likelihood ratio (L) = 16.3; *P *<* *0.001) than fish of the same age in the pre‐collapse years (1987 and 1990), while 2‐year‐old perch were 10.9 mm longer in the post‐collapse years (*L* = 11.8, *P *<* *0.001). As shown in Fig. 2C, most of the difference in juvenile length‐at‐age can be attributed to lower growth rates during the first year of age. No significant differences in length at the ages of 3, 4 or 5 years were found between the pre‐ and post‐collapse groups (group indicator could be reduced from the model with *P *>* *0.05).

In the pre‐collapse samples, 40% of 2‐year old, 37% of 3‐year old and 10% of 4‐year old males were immature, while in the post‐collapse group all the studied males at the ages of two (*n* = 22), three (*n* = 54) and four (*n* = 33) were mature. In males, the probability of being mature (maturity ogive) was found to differ significantly between the pre‐ and post‐collapse groups (*χ* ^2^=−29.9, *P *<* *0.001) as well as to depend on the age of the individual (*χ* ^2^=−17.2, *P *<* *0.001; slope estimate was 0.8193 (*z* = 3.862, *P *<* *0.001)). In females, on the other hand, the probability of being mature did not differ between the pre‐ and post‐collapse groups (*χ* ^2^=−0.262, *P *=* *0.608), but was significantly affected by age (*χ* ^2^=−146.2, *P *<* *0.001; slope estimate was 1.9370 (*z* = 7.754, *P* < 0.001)).

### Deviations from Hardy–Weinberg and gametic phase equilibria

Hardy–Weinberg (HW) equilibrium testing over all loci indicated that five out of 14 cohort samples from Matsalu Bay (1987, 1995, 2006, 2007 and 2008) deviated significantly from HW equilibrium proportions (*P *<* *0.05 before Bonferroni correction). However, after applying the Bonferroni correction, deviations in three recent cohorts (2006–2008) remained significant (Table 1). The linkage disequilibrium test indicated that in the post‐collapse cohorts (2006–2008) a large proportion of locus pairs significantly deviated from the gametic phase equilibrium (*P *<* *0.05; 7–10 locus pairs of 21 comparisons after the Bonferroni correction; Table 1). In contrast, just one locus pair deviated from gametic phase disequilibrium in eleven of the Matsalu Bay cohorts sampled before the population collapse.

### Temporal patterns of genetic diversity

**Figure 3:**
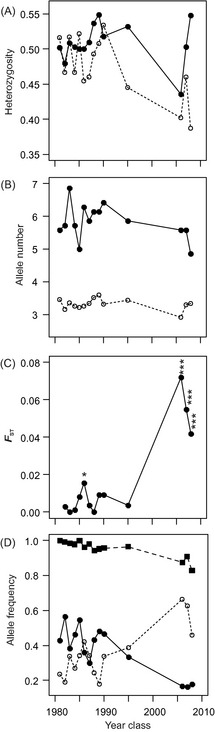
Fluctuations in genetic diversity, differentiation and allelic frequency estimates among perch (*Perca fluviatilis* L.) year classes from Matsalu Bay (1981 to 2008). (A) Expected (*H*_e_) and observed heterozygosity (*H*_o_) (filled and empty circles respectively); (B) mean number of alleles (*A*) and allelic richness (*A*_r_) (filled and empty circles respectively); (C) pairwise *F*_ST_ values between 1981 and later year classes (*P <* *0.05, ***P <* *0.001); (D) frequency estimates of three most variable alleles of microsatellite loci (PFE01‐266: squares, Svi6‐126: filled circles, PFE03‐141: empty circles).

Genetic diversity measures (*H*_o_ *, H*_e_ *, A*,* A*_r_) from Matsalu Bay showed considerable fluctuations over the period of 24 years – the largest changes, however, were associated with the more recent post‐collapse cohorts born in 2006–2008 (Fig. 3). Both mean expected heterozygosity (*H*_e_) and allelic richness (*A*_r_) were lowest in the 2006 cohort, whereas the mean number of alleles (*A*) and observed heterozygosity (*H*_o_) had the smallest values in the 2008 year class (Fig. 3A,B). M‐ratio estimates, on the other hand, showed no marked decrease in post‐collapse cohorts (Table 1), suggesting that a sharp decrease in population size did not result in obvious signs of genetic bottleneck.

### Population differentiation

Estimates of *F*_ST_ between the 1981 and subsequent pre‐collapse year classes (1982–1995) showed relatively stable allele frequencies over 16 years in Matsalu Bay (*F*_ST_ = 0–0.016; most pairwise comparisons non‐significant) (Fig. 3C; see Appendix S1 in Supporting Information). When the earliest cohort (1981) was compared with the post‐collapse cohorts (2006–2008), a sharp increase in genetic differentiation was evident (*F*_ST_ = 0.042–0.072; all pairwise comparisons highly significant, *P *<* *0.001; Fig. 3C). A similar increase in genetic differentiation was observed when cohorts from Matsalu Bay were compared with samples collected from adjacent sea areas (Pärnu versus Matsalu pre‐collapse year classes *F*_ST_ = 0–0.015; most pairwise comparisons non‐significant; Pärnu versus Matsalu post‐collapse year classes *F*_ST_ = 0.038–0.065; all pairwise comparisons highly significant, *P *<* *0.001). Consistent with the *F*_ST_ analysis over all loci, individual allele frequency estimates reflected large genetic shifts in the post‐collapse samples from Matsalu Bay (Fig. 3D). In contrast to the Matsalu Bay cohorts, comparison of temporal samples from Pärnu Bay collected in 1987 and 2010 showed no significant differences.

### Bayesian clustering and analysis of life‐history traits based on multilocus genotype information

**Figure 4:**
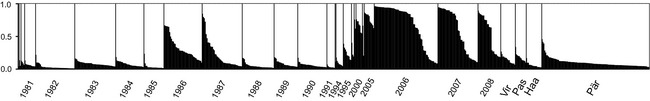
Bayesian clustering analysis using Structure 2.3. Graphical representation of the dataset for the most likely *K *=* *2, where each individual is represented by a vertical bar and the *Y*‐axis represents the probability of assignment of an individual to each cluster. The numbers in the *X*‐axis correspond to a specific cohort (1981–2008) from Matsalu Bay. Nearby samples collected from Virtsu, Paslepa, Haapsalu and Pärnu Bay are indicated as Vir, Pas, Haa, Pär (respectively).

Bayesian clustering analysis showed that the temporal samples from Matsalu Bay and nearby regions can be separated into two distinct genetic groups (Fig. 4). These two clusters were identified using the ΔK method (Evanno et al. 2005) which had highest mean log likelihood of −9886.41 for K2 (−10134.20 = K1, −9941.86 = K3, −9931.12 = K4, −10012.40 = K5 and −10116.90 = K6). This did not distinguish populations based on their geographical proximity but instead, suggested that the majority of post‐collapse individuals spawning in Matsalu Bay (cohorts 1999–2001, 2005–2008) have distinct multilocus genotypes compared to cohorts sampled earlier from the same location. In addition to the post‐collapse samples, a few individuals in the 1986 and 1987 cohorts also possessed multilocus genotypes that resembled post‐collapse samples (Fig. 4).

Analysis of the age‐specific length in post‐collapse cohorts (taking into account the Bayesian clustering information) showed that the individuals belonging to distinct genetic clusters also differed in their growth rates. The length of 1‐year‐old fish depended on sex, inferred cluster and the interaction of sex and inferred cluster (*P *<* *0.05 in all model reductions). The length of 1‐year‐old females carrying the local Matsalu Bay multilocus genotype was on an average 22 mm smaller than for fish carrying a foreign genotype in the post‐collapse sample (SL 70 mm and 92 mm respectively). Similarly, local Matsalu Bay males were smaller than the foreign individuals of the same age (SL 76 mm and 80 mm respectively). At the age of 2 years, the length did not differ between sexes, but perch carrying the local Matsalu genotype were still 16 mm smaller than the individuals of foreign origin (likelihood ratio = 15.2, *P *<* *0.001). At the ages of 3, 4 and 5 years, however, no significant differences between the fishes of local and foreign origin were found (*P *>* *0.05 in all model reductions). In contrast to the analysis of the whole data set (Fig. 2C), only a minor increase in mean length was observed at the age of one year among local fish in the post‐collapse years, when individuals with non‐native genotypes were excluded (Fig. 2D) (likelihood ratio = 5.9, *P *<* *0.015; after Bonferroni correction, *P *>* *0.05). At the ages of 2–5 years, no differences in length between pre‐ and post‐collapse samples with local genotype were found (*P *>* *0.05 in all model reductions).

### Estimation of effective population size and immigration rate

Maximum‐likelihood analyses of effective population size (*N*_e_) and migration rates (*m*) indicated that the estimated *N*_e_ of the Matsalu Bay perch population was small, while the immigration rates, especially in the post‐collapse samples, were high. Consistent with the drastic reduction in census size, the *N*_e_ estimate in the pre‐collapse sample (1981–1990; *N*_e_ = 79, 95% CI = 40–225) was seven times larger than in the post‐collapse sample (1995–2008; *N*_e_ = 11, 95% CI = 7–21). The estimated immigration rate was four times higher in the post‐collapse sample (*m *=* *0.462, 95% CI = 0.296–0.718) compared to the pre‐collapse cohorts (*m *=* *0.114, 95% CI = 0.063–0.181). More detailed cohort‐by‐cohort analysis, however, showed two distinct cases where *m* was much higher than in the other year classes: in 1986 (*m *=* *0.276, 95% CI = 0.157–0.404) and in 2006 (*m *=* *0.264, 95% CI = 0.169–0.317) supporting the results from the Bayesian clustering analysis (Fig. 4).

## Discussion

Both theoretical and empirical studies suggest that intensive fishing can cause changes in the life‐history traits of the harvested population, yet distinguishing possible evolutionary changes from plastic environmental variations is far from trivial (e.g. Stokes and Law 2000; Heino and Godø 2002; Kuparinen and Merilä 2007; Nusslé et al. 2009; Uusi‐Heikkilä et al. 2010; Kuparinen et al. 2011). In this study, we detected marked changes in size‐ and age‐distributions and increases in juvenile growth rates and reductions in age at sexual maturity in males, parameters which have frequently been considered as evidence supporting a FIE scenario. We were not able to estimate probabilistic maturation reaction norms for post‐collapse data as all the fish at age two or older were already mature in contrast to pre‐collapse data, where ca 40% of 2‐ and 3‐year old were immature. However, based on combined individual‐based life‐history and genetic analysis, the findings of this study provide empirical evidence that temporal phenotypic trends can also arise from increased immigration of foreign individuals with different life‐history patterns.

The results of the present study are in accord with recent findings in Atlantic cod, demonstrating that fishing can differentially target genetically and phenotypically distinct components of a population, thus leading to phenotypic changes associated with a change in the population composition (Árnason et al. 2009; Jakobsdóttir et al. 2011). Such genetic shifts would remain unnoticed without the use of molecular genetics tools (Hutchinson et al. 2003) and therefore this study, similar to others (Árnason et al. 2009; Jakobsdóttir et al. 2011), demonstrates the value of combined genetic and life‐history analyses when studying the effects of population recovery and harvesting‐induced selection. When analysing the possible causes behind the changes in life‐history traits, it is therefore important to rule out population replacement, emigration and immigration scenarios as alternative mechanisms behind phenotypic change (Jakobsdóttir et al. 2011; Hansen et al. 2012). The dispersal of individuals may also be non‐random in relation to genotype, individual state and environmental factors (Bowler and Benton 2005; Edelaar and Bolnick 2012). Nevertheless, further research is needed to explicitly test for the presence of non‐random dispersal and gene flow in perch in Matsalu Bay. Currently, there exists a rather limited number of temporal genetic studies of exploited marine fishes (e.g. Hutchinson et al. 2003; Nielsen and Hansen 2008; Árnason et al. 2009; Larsson et al. 2010; Palstra and Ruzzante 2010), and it is possible that many extinctions of local fish populations have gone unnoticed and/or undocumented because of population replacements through immigration (Vasemägi et al. 2001, 2005). Future genetic studies using archived material and new genomic tools could shed light onto this critical but underrated issue (Nielsen and Hansen 2008; Palstra and Ruzzante 2010).

This study also provided insights into effective population size associated with the collapse and recovery of a targeted population. In the Matsalu Bay perch population, a drastic reduction in the effective population size was observed, as *N*_e_ in the post‐collapse period (cohorts 1995–2008) was seven times smaller than in the pre‐collapse period (cohorts 1981–1990). Contrary to expectations, this sharp decrease in *N*_e_ did not result in a rapid loss of genetic diversity. This can be explained by the increased influx of non‐native immigrants, which compensated for the expected loss of genetic diversity among the remnant native population. This increased influx of immigrants is not surprising, given that a reduced *N*_e_ can make the local populations more sensitive to immigration, potentially leading to genetic swamping and/or loss of local adaptations (Hutchinson et al. 2003; Pergams and Lacy 2008; Allendorf et al. 2008; Mackie et al. 2010; Edelaar and Bolnick 2012).

Although it is reasonable to assume that increased immigration is directly related to population collapse, cohort‐by‐cohort analysis indicated that similar, but weaker, influxes of non‐local genotypes may also have happened earlier, before the overexploitation of the Matsalu Bay perch population (e.g. 1986: *m *=* *0.276, 95% CI = 0.157–0.404; Fig. 4). It is important to keep in mind, however, that the estimates of *m* are obtained by pooling immigrant genotypes identified with Structure to establish allele frequencies of a putative donor population. Therefore, estimated migration rates should be regarded with caution until identification of the actual source population of immigrants. Analysis of additional four sampling sites indicated that these geographically close coastal areas (Moonsund Archipelago Sea and Pärnu Bay) most likely do not represent the source population(s) of immigrants that were found in Matsalu Bay. We therefore suggest that the immigrant genotypes observed in our study area might originate from near‐shore littoral perch from the River Kasari delta area that flows into Matsalu Bay, while the vast majority of genotypes from the pre‐collapse samples belong to the more open‐water pelagic form. Hence, the observed phenotypic and genotypic changes may be related to expansion or decrease of the spatial shift in the spawning areas of these populations. The occurrence of genetically and phenotypically distinct but geographically close populations in Eurasian perch is also supported by earlier research. For example, lake and river populations may exchange many migrants due to their proximity (Hendry et al. 2002), also perch from littoral and pelagic zones within the same lake can exhibit significant morphological and growth differences (Svanbäck and Eklöv 2002; Bartels et al. 2012) and evidence for stable genetic differentiation at a small spatial scale have been found both in lakes (Gerlach et al. 2001) and in the Baltic Sea (Bergek and Björklund 2009). As a result, we reiterate that despite the uncertainty regarding the origin of the immigrants, migration remains the most plausible mechanism responsible for the sharp change in genetic composition and marked increase in the frequency of observed Hardy–Weinberg and genotypic equilibrium deviations in the post‐collapse samples collected from Matsalu Bay.

Similar to many earlier studies that focused on the consequences of overfishing, this study describes a rapid collapse of a local population most likely as a result of over‐exploitation, followed by a slow recovery despite of the reductions in fishing pressure (Pauly et al. 2002; Myers and Worm 2003; Hutchings and Reynolds 2004; Olsen et al. 2004, 2005; Pauly 2008). Intriguingly, this reflects the current status of many fish stocks that, in spite of reduced fishing pressure, struggle to recover (Hutchings 2000; Stockwell et al. 2003; Hutchings and Reynolds 2004). It has been suggested that the sharp decrease in the abundance and the slow recovery of perch in the Moonsund Archipelago might be at least partly related to the growing cormorant (*Phalacrocorax carbo sinensis*) colony (Vetemaa et al. 2010), but as only small proportion of the cormorant diet consists of perch (6–10%), increased predation by cormorants most likely does not play a major role in the slow recovery of perch in Matsalu Bay. As an alternative explanation, it is possible that the increase of non‐adaptive alleles and the breakdown of locally adapted assemblages may have had a negative effect on the recovery of the Matsalu Bay perch population. This is in line with an increasing number of studies in both marine and freshwater fishes that show marked population‐level differences in ecologically important traits, suggesting that adaptive population differentiation is likely to be more widespread than previously thought (Hauser and Carvalho 2008). On the other hand, dispersal and gene flow can either promote or constrain adaptive divergence through either genetic or demographic routes (Lenormand 2002; Garant et al. 2007). For example, immigration of maladapted individuals may decrease the mean fitness (Storfer 1999), while immigration can have a healing effect that counteracts inbreeding depression in small populations (Ingvarsson and Whitlock 2000). Future research, combining genomic tools and temporal monitoring of life‐history traits of the exploited fish stocks, is clearly needed to further understand the importance of local adaptation in the field of fisheries science and conservation.

## Data Archiving Statement

Data deposited in the Dryad repository: doi:10.5061/dryad.td237

## Supporting information


**Appendix S1.** Pairwise *F*_ST_‐values between cohorts (below diagonal) and significance levels from genic differentiation (exact G) test (above diagonal).
