# Supplementary material for: Genetic and life‐history changes associated with fisheries‐induced population collapse
Source: Evol Appl. 2013 Feb 25;6(5):749–60. doi: 10.1111/eva.12060 (PMC5779128; doi:10.1111/eva.12060)
Supplement: Supplementary file 1 — Appendix S1. Pairwise F ST‐values between cohorts (below diagonal) and significance levels from genic differentiation (exact G) test (above diagonal). [file EVA-6-749-s001.docx]

**Appendix A: Pairwise *F*_ST_-values between cohorts (below diagonal) and significance levels from genic differentiation (exact G) test (above diagonal).**

Note: NS denotes a non-significant and * (*P*<0.05), ** (*P*<0.01), *** (*P*<0.001) significant *F*_ST_-values before Bonferroni corrections.

|  | 1981 | 1982 | 1983 | 1984 | 1985 | 1986 | 1987 | 1988 | 1989 | 1990 | 1995 | 2006 | 2007 | 2008 | Pärnu |
| --- | --- | --- | --- | --- | --- | --- | --- | --- | --- | --- | --- | --- | --- | --- | --- |
| 1981 | - | NS | NS | NS | NS | * | NS | NS | NS | NS | NS | *** | *** | *** | NS |
| 1982 | 0.003 | - | NS | NS | NS | *** | *** | NS | NS | NS | ** | *** | *** | *** | *** |
| 1983 | 0 | 0.008 | - | NS | NS | NS | NS | NS | NS | NS | NS | *** | *** | *** | NS |
| 1984 | 0.001 | 0.001 | 0 | - | NS | NS | NS | NS | NS | NS | NS | *** | *** | *** | NS |
| 1985 | 0.008 | 0.006 | 0 | 0.003 | - | * | * | NS | NS | NS | * | *** | *** | *** | NS |
| 1986 | 0.016 | 0.026 | 0.004 | 0.003 | 0.018 | - | NS | NS | NS | NS | NS | *** | *** | *** | * |
| 1987 | 0.004 | 0.022 | 0.003 | 0 | 0.016 | 0.003 | - | NS | * | NS | NS | *** | *** | *** | *** |
| 1988 | 0 | 0 | 0 | 0 | 0.002 | 0.007 | 0.002 | - | NS | NS | NS | *** | *** | *** | NS |
| 1989 | 0.009 | 0.013 | 0.010 | 0 | 0.007 | 0.018 | 0.012 | 0 | - | NS | NS | *** | *** | *** | NS |
| 1990 | 0.009 | 0.007 | 0.005 | 0 | 0.013 | 0.011 | 0.005 | 0 | 0.002 | - | NS | *** | *** | *** | NS |
| 1995 | 0.004 | 0.029 | 0.005 | 0.008 | 0.020 | 0 | 0.002 | 0.003 | 0.017 | 0.008 | - | *** | *** | *** | NS |
| 2006 | 0.072 | 0.103 | 0.059 | 0.072 | 0.087 | 0.027 | 0.041 | 0.073 | 0.010 | 0.078 | 0.034 | - | *** | *** | *** |
| 2007 | 0.055 | 0.082 | 0.04 | 0.051 | 0.063 | 0.012 | 0.024 | 0.048 | 0.064 | 0.047 | 0.011 | 0.006 | - | *** | *** |
| 2008 | 0.042 | 0.069 | 0.033 | 0.048 | 0.055 | 0.018 | 0.028 | 0.040 | 0.050 | 0.049 | 0.014 | 0.019 | 0.007 | - | *** |
| Pärnu | 0.003 | 0.015 | 0.005 | 0.002 | 0.005 | 0.010 | 0.010 | 0 | 0 | 0.004 | 0.004 | 0.065 | 0.042 | 0.038 | - |
